# Comprehensive Assessment of Pain and Physiological Parameters of Sympathetic Blockade to Accurately Determine the Success of High Thoracic Erector Spinae Plane Block

**DOI:** 10.3390/healthcare13182322

**Published:** 2025-09-16

**Authors:** Seher İlhan, İlknur Hatice Akbudak, Turan Evran, İsmet Çopur, Çağla Erdoğan, Edip Gönüllü

**Affiliations:** 1 Department of Anesthesiology, Reanimation and Algology, Faculty of Medicine, Pamukkale University, 20070 Denizli, Türkiye; ilhakbudak@gmail.com (İ.H.A.); tevran@pau.edu.tr (T.E.); smtcpr7@gmail.com (İ.Ç.); 2 Adult Intensive Care Unit, Recep Tayyip Erdogan University Training and Research Hospital, 53020 Rize, Türkiye; caglaerdogan89@gmail.com; 3 Department of Anesthesiology, Reanimation and Algology, Faculty of Medicine, Bakırçay University, 35620 İzmir, Türkiye; edipgonullu@gmail.com

**Keywords:** erector spinae plane block, chronic pain, sympathetic nervous system, perfusion index, optic nerve

## Abstract

**Background/Objectives**: High thoracic erector spinae plane block (ESPB) is a novel technique for managing chronic radicular and sympathetically mediated pain. Although physiological changes, such as increased skin temperature, perfusion index (PI), and optic nerve sheath diameter (ONSD), are known to reflect ESPB-induced sympatholysis, their predictive value for analgesic success remains unclear. In this context, the objective of this study is to investigate whether these objective indicators of sympathetic blockade are associated with the clinical success of high thoracic ESPB. **Methods**: The sample of this prospective, observational study consisted of 35 adult patients with chronic radicular pain undergoing a high thoracic ESPB procedure. Pre- and post-procedure assessments included bilateral skin temperature, PI, ONSD measurements, and administration of a visual analog scale (VAS). Patients with a greater than 50% decrease in VAS score were deemed responders to the procedure. **Results**: Of the 35 patients, 29 (82.9%) were responders. The patients’ post-procedure ipsilateral skin temperature (*p* < 0.001), PI (*p* = 0.002), and ONSD (*p* < 0.001) values were significantly higher than their pre-procedure values. However, none of these parameters differed significantly between responders and non-responders (*p* > 0.05). There was also no significant correlation between VAS score and changes in PI, ONSD, or skin temperature (*p* > 0.05). **Conclusions**: In conclusion, although high thoracic ESPB resulted in measurable physiological changes suggestive of sympathetic blockade, these changes did not predict clinical analgesic success.

## 1. Introduction

The erector spinae plane block (ESPB) has emerged as a promising regional anesthesia technique for managing both postoperative and chronic pain, including sympathetically mediated pain syndromes [1,2,3,4]. ESPB can modulate pain transmission through both somatic and visceral pathways by delivering local anesthetic to the interfacial plane between the transverse process and the erector spinae muscle [2,5,6,7].

Anatomical and radiological studies have demonstrated that the injected anesthetic may act on the dorsal and ventral branches of the spinal nerves, including, in some cases, the paravertebral space and sympathetic chain, thereby providing potential analgesic effects for visceral and neuropathic pain [1,3]. However, the precise mechanisms underlying ESPB-induced pain relief are not yet fully understood [8,9].

The efficacy of ESPB is evaluated based on expected outcomes, including pain reduction, functional improvement, and higher quality of life [10]. A narrative review on the role of ESPB in nonsurgical pain management reported that the efficacy of ESPB is most frequently evaluated based on changes in pain scores and patient-reported pain outcomes [11]. Predicting post-interventional pain reduction is essential for tailoring individualized pain management strategies, particularly for patients with chronic low back pain or other persistent pain conditions. High thoracic ESPB, typically performed at the T2–T3 level, has been reported to produce extensive cranio-caudal spread, sometimes reaching from C4 to T10 [3]. This spread occurs via the costotransverse foramina into the ventral ramus, the dorsal ramus, the paravertebral space, and even the contralateral side paravertebral space, including the branching spinal nerve, intercostal vessels embedded in adipose tissue, and sympathetic nerve fibers [3,12,13]. The extent of this distribution depends on both anatomical factors and the volume and concentration of the local anesthetic used [14]. Such unexpected and extensive cranio-caudal coverage may explain the block’s potential efficacy in various chronic pain conditions, including cervical radiculopathy and selected cases of low back pain when performed at higher thoracic levels [12,13].

The anesthetic may exert a sympatholytic effect as it reaches the paravertebral or epidural space, resulting in vasodilation, decreased peripheral vascular resistance, and increased cutaneous blood flow in the corresponding dermatomes [3,4]. Such sympathetic blockade may not only contribute to analgesia but also serve as a surrogate marker of successful block performance. Sympathetic blockade contributes to analgesia through several mechanisms, including attenuation of sympathetically maintained pain, improvement of regional blood flow, reduction in neurogenic inflammation, and interruption of nociceptive–autonomic reflexes [15]. These effects are particularly relevant in conditions where sympathetic overactivity sustains or amplifies pain. Therefore, it has been proposed that sympathetic fiber blockade by local anesthetics during ESPB may serve as a reliable and objective early indicator of block success [16,17].

Skin temperature has long been considered a simple marker of sympathetic activity, as sympathetic blockade results in peripheral vasodilation and an increase in cutaneous blood flow [14,18]. However, temperature-based assessment is susceptible to external environmental influences and may lack sensitivity in detecting subtle or transient changes in sympathetic tone [18].

The perfusion index (PI) is a parameter derived from photoplethysmographic signals obtained via pulse oximetry, which indirectly reflects local perfusion and vasomotor tone [3,7,19]. PI is increasingly being utilized due to its ability to sensitively and promptly indicate sympatholytic effects following epidural blocks and interventions targeting the sympathetic nervous system [18,19,20]. In this regard, PI has emerged as a more advantageous assessment tool compared to temperature-based methods. However, data on potential changes in PI following high thoracic ESPB remain limited.

Additionally, the literature reports that cervical sympathetic block may lead to increased cerebral blood flow, which can, in turn, affect intracranial pressure (ICP) [4]. These physiological changes may include elevated capillary pressure, increased cerebrospinal fluid production, and a subsequent rise in ICP [21].

The optic nerve sheath diameter (ONSD), measured via ultrasonography, is a non-invasive marker of ICP and has been utilized to evaluate such physiological effects [4]. While ONSD is known to change significantly following central blocks such as spinal anesthesia, it is generally believed to remain unaffected by peripheral nerve blocks [22]. Nevertheless, specific procedures—including caudal block [22,23], interlaminar epidural injection [24], and brachial plexus block [25]—have been associated with significant increases in ONSD.

Therefore, standardized, objective, and reproducible methods are needed to evaluate sympathetic blockade and its relationship to the clinical efficacy of ESPB. In this context, parameters such as skin temperature, PI, and ONSD may provide useful objective markers of sympathetic modulation and block success.

In view of the foregoing, the objective of this study is to investigate the relationships between objective indicators of sympathetic blockade and the success of ESPB, defined by a clinically meaningful reduction in post-interventional pain outcomes.

## 2. Materials and Methods

### 2.1. Study Design

This study was designed as a prospective, observational, single-center study. The study protocol was approved by the institutional ethics committee (Approval Number: E-60116787-020-556405, Approval Date: 24 July 2024). The study was conducted at the Pain Management Outpatient Clinic, Department of Anesthesiology and Intensive Care, Faculty of Medicine, Pamukkale University, Denizli, Turkey, between 1 August 2024, and 30 March 2025, in accordance with the ethical considerations outlined in the Declaration of Helsinki. Written informed consent was obtained from all participants prior to the conduct of the study.

### 2.2. Population and Sample

The study population consisted of patients aged 18 to 65 years with chronic radicular pain undergoing ultrasound (US)-guided high thoracic ESPB procedure. Patients with neck pain, with or without arm pain, due to foraminal stenosis, cervical spondylotic myelopathy, or a herniated disk, as well as those with anterior chest or shoulder pain due to herpes zoster or complex regional pain syndrome, were included in the study [7]. In contrast, patients with spinal deformities, history of cervical spine surgery, chronic neurological and visual disorders, coagulation abnormalities, an American Society of Anesthesiologists (ASA) physical status of III or IV as per the official ASA Physical Status Classification System—defined as class III: severe systemic disease with substantive functional limitations, and class IV: severe systemic disease that is a constant threat to life [26], pregnancy, history of an allergic reaction to local anesthetic medications, and bilateral high thoracic ESPBs were excluded. Ultimately, the study sample comprised 35 patients. Patients with a >50% decrease in VAS score were deemed responders to the procedure. Accordingly, 29 (82.9%) patients with a >50% decrease in VAS score were included in the responders group, and 6 (17.1%) patients with a ≤50% decrease in VAS score were included in the non-responders group [7].

### 2.3. Data Collection and Assessments

Patients’ demographic characteristics (age, gender, weight, height, and body mass index [BMI]) and clinical characteristics (pain duration and injection side) were recorded.

Patients were instructed to refrain from smoking, consuming alcohol, and engaging in strenuous physical activity for at least one hour before being evaluated for indicators that potentially reflect ESPB-induced sympatholysis. Additionally, they were asked to remove all jewelry on their hands or fingers to minimize interference with pulse oximetry readings and prevent potential artifacts in photoplethysmographic signal acquisition. They were then placed in a room with an ambient temperature between 24 °C and 26 °C, free from all potentially confounding external factors, including any heat-generating equipment.

Patients’ vital signs, including heart rate, oxygen saturation (SpO_2_), and blood pressure, were measured using non-invasive monitors (pulse oximetry and blood pressure cuff) after they had rested for at least 10 min [7]. Skin temperature was measured at the volar aspect of the index fingers on both the affected ipsilateral and contralateral sides using a touch thermometer (Patient Monitor VM 8; Device Registration Number: US 12563255, Philips Inc., Amsterdam, The Netherlands). After body temperature readings stabilized within five minutes, the last reading was recorded as the baseline skin temperature.

Baseline pain intensity was assessed using the visual analog scale (VAS), scored from 0 (no pain) to 10 (worst imaginable pain) [27].

The baseline perfusion index (PI) was measured at the index or third fingertip of each hand using a Masimo^®^ pulse oximetry sensor (Rad-97, Pulse CO-Oximeter, Device Registration Number: 3000212636, Masimo Corp., Irvine, CA, USA) [19].

For optic nerve sheath diameter (ONSD) measurements, patients were placed in the supine position. A 3.5–10.0 MHz wide-band linear array probe (LOGIQ e Ultrasound, Device Registration Number: 504777WX4, GE Healthcare, Wauwatosa, WI, USA) was gently placed over the patients’ closed upper eyelids. The optic nerve was visualized in 2D mode, and the diameter was measured 3 mm posterior to the globe using electronic calipers. Two measurements were made for each eye, in the horizontal and vertical planes. The two measurements were averaged to obtain the ONSD for both affected and contralateral sides [4].

### 2.4. High Thoracic ESPB Procedure

**Figure 1:**
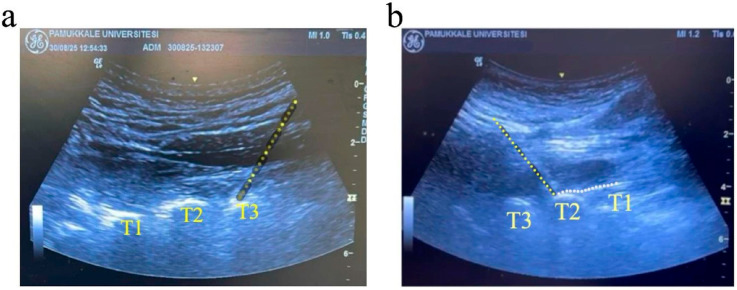
Ultrasound images of high thoracic ESPB at the T2-T3 level. (**a**) Ultrasound image showing the T1, T2, and T3 transverse processes with the erector spinae muscle overlying these structures. The yellow dotted line indicates the needle path. (**b**) Ultrasound image demonstrating local anesthetic spread (white dotted line) beneath the erector spinae muscle fascia at the T2–T3 level.

All ESPB procedures were performed by a single experienced physician with more than 10 years of experience in US-guided regional anesthesia. The injection side was determined based on the patient’s pain location. Patients were positioned in either the sitting or the prone position, whichever they felt comfortable in. A high-frequency linear probe (Logiq™ S8, GE Healthcare, Wuxi, Jiangsu, China) was inserted longitudinally within a sterile sheath. After locating the spinous process, lamina, and T2 or T3 transverse process, a 100 mm, 22-gauge echogenic needle was advanced in a cephalad-to-caudal plane to contact the transverse process. After confirming that the needle was correctly placed, 20 mL of 0.2% ropivacaine solution was injected, and a linear spread of the local anesthetic beneath the erector spinae muscle was confirmed by US (Figure 1) [7]. Thirty minutes after the ESPB procedure, vital signs, including skin temperature at the ipsilateral affected and unaffected contralateral sides, VAS scores, PI, and ONSD values, were re-evaluated. Any adverse effects, such as Horner syndrome, flushing, nasal obstruction, or dysphonia that developed after the ESPB procedure were documented [7].

### 2.5. Sample Size Calculations

Sample size calculations were performed using G*Power 3.1.9.6 software based on Hong et al.’s study [7], which reported a 64% success rate for the ESPB procedure. Based on α = 0.05 and 80% power, the minimum number of patients required was calculated to be 114 using Fisher’s exact test for two independent rates, specifically 0.64 vs. 0.36. Given the exploratory nature of this study, which aims to investigate novel physiological parameters in high thoracic ESPB, we conducted a pilot study involving 35 patients. The observed treatment response rate of 82.9% exceeded our anticipated rate of 80%.

### 2.6. Statistical Analysis

The primary endpoint was procedural success, defined as a greater than 50% reduction in VAS pain score at 30 min post-procedure. Secondary endpoints included the association between skin temperature, PI, and ONSD, as well as procedural success, and correlations between these physiological parameters and analgesic outcomes.

Jamovi project 2.6.44 (Jamovi, version 2.6.44, 2025, retrieved from https://www.jamovi.org accessed on 1 July 2025) and JASP 0.19.3 (Jeffreys’ Amazing Statistics Program, version 0.19.3, 2025, retrieved from https://jasp-stats.org accessed on 1 July 2025) software packages were used in the statistical analyses of the collected data.

The results of the statistical analyses were expressed using descriptive statistics, i.e., median values with minimum and maximum values in the case of non-normally distributed continuous variables, and frequencies (*n*) and percentages (%) in the case of categorical variables. The normal distribution characteristics of numerical variables were analyzed using the Shapiro–Wilk test and visual tools, such as histograms and Q-Q plots. The Mann–Whitney U test was used to compare the differences in non-normally distributed continuous variables between the responders and non-responders groups, and Fisher’s exact test was used to compare the differences in categorical variables, including gender, the injection side, and adverse effects, given the small size of the non-responder group.

The comparisons of pre- and post-procedure hemodynamic parameters, body temperature, ONSD, and PI within the study groups were made using the Wilcoxon signed-rank test for paired data, which accounted for the dependent nature of repeated measurements from the same individuals.

Percentage changes in PI were calculated using the following formula: [(PI at time point − PI at baseline)/PI at baseline] × 100. These derived variables were compared between the groups using the Mann–Whitney U test.

Probability (*p*) statistics of ≤0.05 were deemed to indicate statistical significance.

## 3. Results

The distribution of patients’ baseline demographic and clinical characteristics by study group is presented in Table 1. Accordingly, there were no significant differences between the study groups in terms of age, gender, BMI, or injection side (*p* > 0.05).

The distribution of patients’ pre- and post-procedure hemodynamic characteristics and VAS scores by the study groups is given in Table 2. Accordingly, the median baseline systolic blood pressure of the non-responder group was significantly higher than that of the responder group (*p* = 0.042). The median systolic blood pressure increased in both groups, although the change was not statistically significant (*p* = 0.110). The median heart rate decreased significantly in the responder group (*p* = 0.024). There was no significant change in SpO_2_ in either group (*p* > 0.05). Ipsilateral body temperature increased significantly in both groups (*p* < 0.001 for responders and *p* = 0.028 for non-responders), while contralateral body temperature increased significantly in the responder group only (*p* < 0.001).

The sample’s median post-procedure VAS score of 2.0 (1.0 to 8.0) was significantly lower than the median pre-procedure VAS score of 8.0 (6.0 to 10.0) (*p* < 0.001). The median VAS scores decreased significantly in each study group, specifically from 8.0 to 2.0 in the responder group and from 6.0 to 3.0 in the non-responder group (*p* < 0.05).

The distribution of sonographic characteristics of the optic nerve sheath by the study groups is given in Table 3. Accordingly, horizontal, vertical, and average ipsilateral ONSDs increased significantly overall and in each study group (*p* < 0.001). On the other hand, horizontal and average contralateral ONSDs increased significantly in the responder group only (*p* = 0.001 and *p* = 0.002, respectively). In other words, the grouping based on treatment success did not affect the pre- and post-procedure ONSD measurements (*p* > 0.05).

The distribution of PI measurements and percentage changes thereof by the study groups is given in Table 4. Accordingly, the median ipsilateral PI of the sample increased from 2.6 before the procedure to 3.0 30 min after the procedure (*p* = 0.002). The median ipsilateral PI also increased significantly in the responders and non-responders groups (*p* = 0.022 and *p* = 0.036, respectively). The percentage change in ipsilateral PI at 30 min after the procedure, compared to before the procedure, was higher, albeit not significantly, in the non-responder group than in the responder group (29.9% vs. 11.1%, *p* = 0.347). In other words, the grouping based on treatment success did not affect the pre- and post-procedure PI measurements (*p* > 0.05).

Correlation analyses revealed no significant correlations between VAS score, skin temperature, PI, and ONSD at the ipsilateral and contralateral sides (*p* > 0.05) (Table 5).

The distribution of adverse effects experienced by patients after the procedure by the groups is given in Table 6. Accordingly, the incidence of adverse effects in the sample was 48.6%. Facial flushing (34.3%) and nasal congestion (20.0%) were the most frequently reported symptoms, followed by Horner syndrome (5.7%) and hoarseness (2.9%). There were no significant differences in adverse effect rates between the groups (*p* > 0.05).

## 4. Discussion

The study’s findings indicated significant increases in ipsilateral skin temperature, PI, and ONSD in both the responder and non-responder groups following ESPB, with no significant difference between the groups, suggesting that their utility as standalone indicators of ESPB success may be limited. The lack of a significant correlation between PI and ONSD measurements suggests that distinct mechanisms may underlie the increases in these measurements after the procedure. These findings emphasize both the localized and systemic physiological effects of high thoracic ESPB and underscore the complexity of interpreting concurrent changes in vascular and neurologic parameters, reinforcing the importance of adopting a multimodal approach when evaluating ESPB efficacy, rather than relying on any single monitoring parameter.

If these physiological markers prove to be reliable predictors of block effectiveness, they could substantially improve clinical decision-making by providing objective, minimally invasive, and real-time evidence of sympathetic blockade. Early identification of non-responders would allow timely modifications such as block supplementation, adjustment of anesthetic dosage, or transition to alternative analgesic strategies. This proactive approach could minimize patient discomfort, reduce the risks associated with repeated ineffective interventions, and optimize resource utilization. Moreover, linking the degree of sympathetic blockade with anticipated pain relief enables individualized treatment planning. Predicting post-intervention pain reduction is clinically important because it allows analgesic strategies to be adapted before breakthrough pain occurs, thereby improving patient comfort and safety. Linking the degree of sympathetic blockade with anticipated pain relief facilitates individualized treatment planning. For example, patients showing robust sympathetic blockade could be prioritized for early mobilization and physiotherapy. At the same time, those demonstrating minimal physiological changes might benefit from preemptive multimodal analgesia, closer monitoring, or psychological support. In this way, physiological markers provide not only a practical tool for block evaluation but also a pathophysiologically grounded basis for optimizing post-interventional care.

The presence of sympathetic blockade during peripheral nerve blocks can be interpreted as an indicator of effective anesthetic spread and potentially enhanced analgesia; however, it does not necessarily predict the success of interventional pain procedures. Indeed, in a comprehensive review of whether sympathetic blockade could predict successful intervention, Raja and Treede [16] concluded that the diagnostic value of sympathetic blocks for identifying sympathetically maintained pain was inconsistent and limited. Similarly, Vadhanan et al. [17] noted that sympathetic blockade is primarily a physiological consequence of local anesthetic action on autonomic fibers. They emphasized that although it may contribute to analgesia through vasodilation and modulation of reflex arcs, its analgesic effect is multifactorial due to factors such as somatic afferent interruption, central desensitization, and autonomic modulation; thus, isolating sympathetic effects as prognostic markers is difficult.

Although traditional clinical signs, including Horner’s syndrome, Harlequin syndrome, and changes in skin temperature, are frequently used as indicators of sympathetic blockade, their predictive value is limited [3,4,14,18,19,28,29]. Stevens et al. [18] proposed using three criteria to evaluate sympatholysis after a stellate ganglion block: a temperature difference of ≥1.5 °C between the ipsilateral and contralateral sides, the presence of Horner’s syndrome, and conversion of a positive sweat test result to a negative one. However, nearly half of the patients in their sample failed to meet all three criteria. Furthermore, stellate ganglion block often failed to elicit a greater temperature increase on the ipsilateral hand compared to the contralateral side.

The quest for reliable, non-invasive markers of sympathetic blockade has therefore focused on parameters such as PI and ONSD [3,4,7,19]. Rather than reiterating their physiological basis, our results emphasize their clinical behavior after ESPB [30,31]. We found that PI values increased significantly after ESPB; however, we did not find a significant correlation between these changes and decreased pain severity, which is consistent with the findings of previous studies that reported PI increased after ESPB but did not provide meaningful pain relief for all patients [3,7]. Similarly, Chung et al. [19] reported that PI changes may not consistently correlate with clinical outcomes, particularly in patients with pain featuring mixed neuropathic and sympathetically maintained components. ONSD, widely investigated as a surrogate marker of intracranial pressure, also increased significantly after ESPB in our cohort [4,22,25,32]. Some studies have reported increases in OND following sympathetic blockade [4,22,23,24,25]; however, the clinical relevance and reproducibility of this finding remain uncertain [32]. However, similar to PI, these changes did not correlate with decreased pain severity, suggesting that ONSD primarily reflects physiological response rather than analgesic efficacy. This observation aligns with previous reports that describe increases in ONSD after sympathetic blockade or neuraxial techniques [3,7,19,23].

Although we observed increases in ipsilateral temperature following ESPB, we did not find any significant relationship between skin temperature and treatment success, in line with the findings of Stevens et al. [18], who reported that approximately half of their patients failed to meet their proposed temperature-based criteria to evaluate sympatholysis after stellate ganglion block, suggesting that rigid temperature thresholds may be overly stringent and insufficiently sensitive as clinical predictors.

The persistence of pain in non-responders may reflect underlying pathophysiological changes associated with chronic nerve compression. Prolonged mechanical compression can cause intraneural fibrosis, demyelination, and axonal degeneration, leading to both peripheral and central sensitization. Schmid et al. [33] demonstrated an apparent reduction in intraepidermal nerve fiber density and a disturbed nodal structure/myelin well beyond the focal compression site in patients with entrapment neuropathies. As these neuroplastic changes progress, neuropathic pain mechanisms may become predominant, diminishing the contribution of sympathetically maintained components and thereby reducing the effectiveness of sympathetic blockade. This finding provides new insight into patient stratification: physiological markers of sympathetic activity may help distinguish individuals likely to benefit from sympathetic blocks from those who may require alternative analgesic strategies, such as multimodal pharmacotherapy, neuromodulation, or psychological interventions. In this way, our study extends the observations of Raja et al. [16] and Vadhanan et al. [17] by suggesting that objective physiological markers can complement pain assessments, offering an earlier and more pathophysiologically grounded method for evaluating block effectiveness in the context of chronic pain. Despite the extensive current literature on this topic, we still do not fully understand how we recognize pain and itch as distinct sensations with different qualities in the same context of chronic sensitization. Literature suggests that both chronic pain and itch may share strikingly similar underlying mechanisms. Especially, peripheral and central sensitization lead to the development and persistence of chronic dysesthesias [34]. The similarities between chronic itch and pain suggest the need to combine studies investigating both itch and pain, thereby facilitating the development of new therapeutics against both chronic dysesthesias.

Taken together, our findings reinforce that autonomic changes following ESPB are complex and multifactorial. While PI, ONSD, and skin temperature offer objective evidence of physiological responses to ESPB, their ability to predict analgesic success remains limited. In other words, some patients may experience sympathetic blockade without a significant analgesic effect, while others may not, depending on the underlying mechanisms of their pain. The observed dissociation between physiological and clinical responses may be attributed to variations in the spread of local anesthetics, individual anatomy, or differential modulation of somatic and sympathetic fibers.

If these physiological markers prove to be reliable predictors of block effectiveness, they could substantially improve clinical decision-making. Early identification of non-responders would allow timely adjustments such as block supplementation, dose modification, or switching to alternative analgesic strategies. This proactive approach could minimize patient discomfort, reduce the risks associated with repeated interventions, and improve resource efficiency. Furthermore, anticipating post-interventional pain reduction through objective physiological markers facilitates individualized treatment planning. For example, patients demonstrating robust sympathetic blockade could be prioritized for early mobilization and physiotherapy, while those showing minimal changes might benefit from preemptive multimodal analgesia or psychological support.

Unlike previous studies by Raja and Treede et al. [16] or Vadhanan et al. [17], which mainly addressed theoretical aspects of sympathetic blockade, our study provides prospective clinical data comparing multiple objective physiological parameters with patient-reported outcomes. By evaluating PI, ONSD, and skin temperature simultaneously during high thoracic ESPB, we provide the first systematic attempt to correlate sympathetic markers with analgesic success in this block. These markers reflect autonomic changes immediately after the intervention, allowing earlier detection of block efficacy than pain scores alone, and underscore the clinical relevance of sympathetic modulation in chronic pain management.

Our findings support the argument that no single physiological marker can reliably predict ESPB success, and that a multimodal approach combining subjective observations such as reported pain relief, warmth, and color changes with objective metrics such as PI, skin temperature, and ONSD, and incorporating additional tools such as sympathetic skin response or Doppler-based perfusion imaging may be more effective [3,4,19].

This study had several limitations. First, the limited sample size, particularly the small number of patients in the non-responders group, may limit the study’s statistical power. Secondly, the fact that a single team performed all procedures at a single center may affect the generalizability of the study’s findings. Thirdly, although we used objective markers, the fact that we did not employ other valuable techniques, such as laser Doppler flowmetry, sweat testing, or direct imaging of injectate spread, may be considered another limitation of the study. Fourthly, the fact that we used ONSD as a surrogate marker of intracranial pressure, without direct measurement, and without blinding the assessments, may have introduced bias. Lastly, the absence of long-term follow-up may limit the ability to correlate early physiological responses with sustained pain relief.

## 5. Conclusions

In conclusion, high thoracic ESPB resulted in significant increases in PI, ONSD, and skin temperature, confirming its autonomic and physiological impact. However, these changes were not predictive of analgesic success. Our findings underscore the importance of a multimodal assessment strategy that integrates clinical evaluation with objective physiological monitoring to establish ESPB efficacy and enhance patient outcomes. Future studies with larger cohorts and broader autonomic profiling are needed to clarify the predictive roles of these physiological markers in interventional pain management.

## Figures and Tables

**Table 1:** Baseline demographic and clinical characteristics of patients undergoing high thoracic erector spinae plane block stratified by treatment response.

Variables	Overall (n = 35)	Responders (VAS > 50%) (n = 29)	Non-Responders (VAS ≤ 50%) (n = 6)	*p*
**Age** (years)	53.0 [27.0–79.0]	49.0 [27.0–79.0]	55.0 [44.0–67.0]	0.380 *
**Sex**				
Female	19 (54.3)	16 (55.2)	3 (50.0)	0.999 **
Male	16 (45.7)	13 (44.8)	3 (50.0)	
**Body mass index** (kg/m^2^)	26.2 [20.8–33.3]	26.2 [20.8–33.3]	29.1 [21.3–31.6]	0.418 *
**Pain duration** (months)	1.0 [1.0–2.0]	1.0 [1.0–2.0]	1.5 [1.0–2.0]	0.839 *
**Side**				
Right	18 (51.4)	15 (51.7)	3 (50.0)	0.999 **
Left	17 (48.6)	14 (48.3)	3 (50.0)	

Data are presented as median [minimum–maximum] for continuous variables and n (%) for categorical variables. Treatment response was classified based on visual analog scale (VAS) reduction, with responders defined as patients achieving >50% reduction and non-responders as those with ≤50% reduction. * Mann–Whitney U test; ** Fisher’s exact test.

**Table 2:** Hemodynamic parameters and pain scores following high thoracic erector spinae plane block.

Variables	Responders (VAS > 50%) (n = 29)	Non-Responders (VAS ≤ 50%) (n = 6)	*p*
**Heart rate** (beats/min)			
Pre-procedure	79.0 [65.0–100.0]	84.5 [70.0–98.0]	0.381 *
Post-procedure 30 min	79.0 [66.0–93.0]	78.5 [73.0–94.0]	0.456 *
*p* **	0.131	0.999	
**SpO_2_** (%)			
Pre-procedure	98.0 [95.0–100.0]	96.5 [95.0–99.0]	0.168 *
Post-procedure 30 min	99.0 [95.0–100.0]	96.5 [95.0–99.0]	0.136 *
*p* **	0.157	0.999	
**SBP** (mmHg)			
Pre-procedure	124.0 [93.0–166.0]	137.5 [127.0–163.0]	**0.042** *
Post-procedure 30 min	130.0 [90.0–165.0]	142.0 [128.0–159.0]	0.110 *
*p* **	0.075	0.245	
**DBP** (mmHg)			
Pre-procedure	63.0 [50.0–94.0]	77.5 [52.0–97.0]	0.368 *
Post-procedure 30 min	67.0 [50.0–91.0]	66.0 [54.0–95.0]	0.792 *
*p* **	0.086	0.917	
**Ipsilateral skin temperature** (°C)			
Pre-procedure	34.7 [33.4–35.8]	34.4 [33.9–35.5]	0.693 *
Post-procedure 30 min	36.0 [34.5–36.5]	36.5 [35.3–36.6]	0.186 *
*p* **	**<0.001**	**0.028**	
**Contralateral skin temperature** (°C)			
Pre-procedure	34.6 [33.4–36.0]	34.2 [33.9–35.7]	0.660 *
Post-procedure 30 min	34.8 [33.5–36.2]	34.2 [34.0–35.7]	0.510 *
*p* **	**<0.001**	0.498	
**VAS**			
Pre-procedure	8.0 [6.0–10.0]	6.0 [6.0–9.0]	0.054 *
Post-procedure 30 min	2.0 [1.0–3.0]	3.0 [3.0–8.0]	**NA**
*p* **	**<0.001**	**0.024**	

Data are presented as median [minimum–maximum]. SpO_2_: Peripheral oxygen saturation; SBP: Systolic blood pressure; DBP: Diastolic blood pressure; VAS: Visual analog scale; NA: Not applicable. * Mann–Whitney U test for between-group comparisons; ** Wilcoxon signed-rank test for within-group comparisons. Bold *p*-values indicate statistical significance (*p* ≤ 0.05).

**Table 3:** Sonographic measurements of optic nerve sheath diameter and laterality of procedure in patients undergoing high thoracic erector spinae plane block.

Variables	Overall (n = 35)	Responders (VAS > 50%) (n = 29)	Non-Responders (VAS ≤ 50%) (n = 6)	*p*
**Ipsilateral optic nerve** (mm, horizontal)				
Pre-block	0.47 [0.36–0.65]	0.48 [0.38–0.65]	0.41 [0.36–0.61]	0.254 *
Post-block	0.51 [0.43–0.69]	0.53 [0.43–0.69]	0.48 [0.45–0.65]	0.262 *
*p* **	**<0.001**	**<0.001**	**0.027**	
**Ipsilateral optic nerve** (mm, vertical)				
Pre-block	0.46 [0.36–0.65]	0.46 [0.38–0.65]	0.40 [0.36–0.61]	0.227 *
Post-block	0.52 [0.43–0.68]	0.52 [0.43–0.68]	0.51 [0.45–0.64]	0.999 *
*p* **	**<0.001**	**<0.001**	**0.028**	
**Average ipsilateral optic nerve** (mm)				
Pre-block	0.47 [0.36–0.65]	0.47 [0.38–0.65]	0.40 [0.36–0.61]	0.228 *
Post-block	0.51 [0.44–0.69]	0.52 [0.44–0.69]	0.50 [0.45–0.64]	0.584 *
*p* **	**<0.001**	**<0.001**	**0.031**	
**Contralateral optic nerve** (mm, horizontal)				
Pre-block	0.47 [0.39–0.65]	0.47 [0.39–0.65]	0.50 [0.39–0.63]	0.614 *
Post-block	0.48 [0.40–0.65]	0.48 [0.40–0.65]	0.50 [0.40–0.64]	0.692 *
*p* **	**<0.001**	**0.001**	0.102	
**Contralateral optic nerve** (mm, vertical)				
Pre-block	0.49 [0.40–0.62]	0.49 [0.40–0.61]	0.49 [0.40–0.62]	0.861 *
Post-block	0.49 [0.40–0.62]	0.49 [0.40–0.62]	0.49 [0.40–0.62]	0.742 *
*p* **	0.285	0.655	0.317	
**Average contralateral optic nerve** (mm)				
Pre-block	0.48 [0.40–0.63]	0.48 [0.40–0.63]	0.50 [0.40–0.62]	0.693 *
Post-block	0.49 [0.40–0.63]	0.49 [0.40–0.62]	0.50 [0.40–0.63]	0.742 *
*p* **	**<0.001**	**0.002**	0.174	

Data are presented as median [minimum–maximum]. * Mann–Whitney U test for between-group comparisons; ** Wilcoxon signed-rank test for within-group comparisons between pre-block and post-block measurements. Bold *p*-values indicate statistical significance (*p* ≤ 0.05).

**Table 4:** Perfusion index measurements and percentage changes following high thoracic erector spinae plane block.

Variables	Overall (n = 35)	Responders (VAS > 50%) (n = 29)	Non-Responders (VAS ≤ 50%) (n = 6)	*p*
**Ipsilateral perfusion** **index**				
Pre-procedure	2.6 [0.4–7.3]	2.6 [0.4–7.3]	2.3 [1.4–4.5]	0.540 *
Post-procedure 30 min	3.0 [0.7–7.1]	3.0 [0.7–7.1]	3.0 [2.5–4.6]	0.999 *
p **	**0.002**	**0.022**	**0.036**	
**%Δ PI-0/30 min** (ipsilateral)	11.1 [−23.1–100.0]	11.1 [−23.1–100.0]	29.9 [2.2–100.0]	0.347 *
**Contralateral perfusion index**				
Pre-procedure	3.3 [0.5–8.2]	3.1 [0.5–8.2]	4.4 [2.0–8.0]	0.539 *
Post-procedure 30 min	3.5 [0.4–7.9]	3.4 [0.4–7.9]	4.4 [2.0–7.7]	0.483 *
*p* **	0.111	0.073	0.999	
**%Δ PI-0/30 min** (contralateral)	−2.0 [−23.1–46.7]	−2.0 [−23.1–46.7]	−1.0 [−3.8–12.0]	0.479 *

Data are presented as median [minimum–maximum]. %Δ PI: Percentage change in perfusion index calculated as [(PI at 30 min − PI at baseline)/PI at baseline] × 100. * Mann–Whitney U test for between-group comparisons; ** Wilcoxon signed-rank test for within-group comparisons. Bold *p*-values indicate statistical significance (*p* ≤ 0.05).

**Table 5:** Correlation analysis between VAS pain score, skin temperature, perfusion index, and optic nerve sheath diameter measurements.

			VAS Pain Score	
			r	*p*
**Pre-intervention**	**Ipsilateral**	Skin temperature	0.051	0.770
		PI	0.090	0.609
		ONSD	0.295	0.085
	**Contralateral**	Skin temperature	0.075	0.668
		PI	−0.103	0.554
		ONSD	0.259	0.132
**Post-intervention**	**Ipsilateral**	Skin temperature	−0.023	0.896
		PI	−0.074	0.671
		ONSD	−0.253	0.143
	**Contralateral**	Skin temperature	−0.098	0.574
		PI	0.003	0.987
		ONSD	−0.040	0.819

r: Spearman’s correlation coefficient; VAS: visual analog scale, PI: perfusion index; ONSD: optic nerve sheath diameter.

**Table 6:** Adverse effects following high thoracic erector spinae plane block.

Variables	Overall (n = 35)	Responders (VAS > 50%) (n = 29)	Non-Responders (VAS ≤ 50%) (n = 6)	*p* *
**Adverse effects**, present	17 (48.6)	13 (44.8)	4 (66.7)	0.402
**Facial flushing**, present	12 (34.3)	9 (31.0)	3 (50.0)	0.391
**Nasal congestion**, present	7 (20.0)	6 (20.7)	1 (16.7)	0.999
**Horner syndrome**, present	2 (5.7)	2 (6.9)	0 (0.0)	0.999
**Hoarseness**, present	1 (2.9)	1 (3.4)	0 (0.0)	0.999

Data are presented as n (%). For variables marked with present, only the presence of the condition is displayed, as these represent binary present/absent variables. VAS: Visual analog scale. * Fisher’s exact test. Bold *p*-values indicate statistical significance (*p* ≤ 0.05).

## Data Availability

The data presented in this study are not publicly available due to patient confidentiality and institutional ethical requirements but will be shared on reasonable request from the corresponding author.

## Abbreviations

The following abbreviations are used in this manuscript:

ASA	American Society of Anesthesiologists
BMI	Body Mass Index
CI	Confidence Interval
DBO	Diastolic Blood Pressure
ESPB	Erector Spinae Plane Block
GA	Graphical Abstract
ICMJE	International Committee of Medical Journal Editors
IQR	Interquartile Range
IRB	Institutional Review Board
ONSD	Optic Nerve Sheath Diameter
PI	Perfusion Index
SBP	Systolic Blood Pressure
SD	Standard Deviation
SpO_2_	Peripheral Oxygen Saturation
US	Ultrasound
VAS	Visual Analog Scale
